# A Model for a Standardized and Sustainable Pediatric Anesthesia-Intensive Care Unit Hand-Off Process

**DOI:** 10.3390/children7090123

**Published:** 2020-09-03

**Authors:** Priti G. Dalal, Theodore J. Cios, Theodore K. M. DeMartini, Amit A. Prasad, Meghan C. Whitley, Joseph B. Clark, Leon Lin, Dennis J. Mujsce, Robert E. Cilley

**Affiliations:** 1 Departments of Anesthesiology and Peri-Operative Medicine, Penn State Health Milton S Hershey Medical Center, Penn State Health Children’s Hospital, Hershey, PA 17033, USA; tcios@pennstatehealth.psu.edu (T.J.C.); aprasad@pennstatehealth.psu.edu (A.A.P.); mwhitley@pennstatehealth.psu.edu (M.C.W.); 2 Division of Pediatric Critical Care, Department of Pediatrics, Penn State Health Children’s Hospital, Hershey, PA 17033, USA; tdemartini@pennstatehealth.psu.edu; 3 Division of Pediatric Cardiac Surgery, Department of Pediatrics, Penn State Health Children’s Hospital, Hershey, PA 17033, USA; jclark7@pennstatehealth.psu.edu; 4 Department of Emergency Medicine, Ohio State Universirty, Columbus, OH 43210, USA; Lin.leon02@gmail.com; 5 Division of Newborn Medicine, Department of Pediatrics, Penn State Health Children’s Hospital, Hershey, PA 17033, USA; dmujsce@pennstatehealth.psu.edu; 6 Division of Pediatric Surgery, Department of Surgey, Penn State Health Children’s Hospital, Hershey, PA 17033, USA; rcilley@pennstatehealth.psu.edu

**Keywords:** patient hand-off, intensive care, children, anesthesia

## Abstract

Background and Objectives: The hand-off process between pediatric anesthesia and intensive care unit (ICU) teams involves the exchange of patient health information and plays a major role in reducing errors and increasing staff satisfaction. Our objectives were to (1) standardize the hand-off process in children’s ICUs, and (2) evaluate the provider satisfaction, efficiency and sustainability of the improved hand-off process. Methods: Following multidisciplinary discussions, the hand-off process was standardized for transfers of care between anesthesia-ICU teams. A pre-implementation and two post-implementation (6 months, >2 years) staff satisfaction surveys and audits were conducted to evaluate the success, quality and sustainability of the hand-off process. Results: There was no difference in the time spent during the sign out process following standardization—median 5 min for pre-implementation versus 5 and 6 min for post-implementation at six months and >2 years, respectively. There was a significant decrease in the number of missed items (airway/ventilation, venous access, medications, and laboratory values pertinent events) post-implementation compared to pre-implementation (*p* ≤ 0.001). In the >2 years follow-up survey, 49.2% of providers felt that the hand-off could be improved versus 78.4% in pre-implementation and 54.2% in the six-month survey (*p* < 0.001). Conclusion: A standardized interactive hand-off improves the efficiency and staff satisfaction, with a decreased rate of missed information at the cost of no additional time.

## 1. Introduction

Patient “sign-out” or “hand-off” is a critical part of the provision of medical care. This is particularly important with respect to critically ill patients, especially children. A hand-off is defined by The Joint Commission as the “real-time process of passing patient-specific information from one caregiver to another or from one team of caregivers to another for the purpose of ensuring the continuity of patient’s care” [1]. The hand-off process involves the exchange of patient health information, as well as the transfer of patient care and responsibility. A detailed and pertinent hand-off also plays a major role in reducing errors [2], improving outcomes, and increasing staff satisfaction [3]. Studies have shown that intensive care units have a higher rate of medical errors compared to other units in the hospital [4,5]. While there is some literature surrounding the use of checklists and protocols for these hand-off processes with respect to the transition of care from the operating room (OR) to the intensive care unit (ICU) [6], there is not much literature on the transition of care from the ICU to the OR. Further, there is sparse data on the staff satisfaction, sustainability and effectiveness of a standardized hand-off process. 

In our children’s hospital, we identified the variability and lack of a formal hand-off process during OR–ICU and ICU–OR hand-off. We identified anecdotally missed critical information such as failed extubation, difficult intubation, medications administered, abnormal laboratory values (electrolyte abnormalities, coagulation abnormalities, etc.) that could potentially have led to errors in patient care, as well as overall provider dissatisfaction based on anecdotes from providers participating in the hand-off process. As a result, stake holders from anesthesia, surgery and ICU teams (intensivist, nurse, respiratory therapist) of the children’s hospital met to discuss this as a quality improvement (QI) project. The aims of our QI project were to (1) standardize the process of anesthesia-ICU hand-off communication in children’s ICUs and (2) evaluate provider satisfaction, efficiency and sustainability of a standardized hand-off process after the implementation of the process change. 

## 2. Materials and Methods

The Institutional Review Board (IRB) waived the requirement for a review of this QI project.

### 2.1. Setting

This QI project was implemented at our children’s hospital, which is an academic tertiary care center and American College of Surgeons Level 1 verified Children’s Surgical Center. The 75-bed children’s hospital is a part of the main university hospital. It is a dedicated stand-alone building connected to the adult hospital via a bridge. Our children’s hospital encompasses two ICUs, i.e., a 42-bed neonatal intensive care unit (NICU) and an 18-bed pediatric intensive care unit (PICU). The anesthesia team, along with the surgical team, is involved with the transport of critically ill children from the ICU to the OR (Exit ICU) and from the OR to the ICU (Entry ICU). Hence, the transition of care of critically ill children occurs between multiple teams. 

### 2.2. Planning the Intervention 

Prior to the study, there was variability in the hand-off process with a potential for miscommunication, loss of information and provider dissatisfaction. There was a lack of clarity with regard to the start and end of the existent hand-off process, and the point when the onward clinical care responsibility was assumed by the receiving team. 

**Figure 1:**
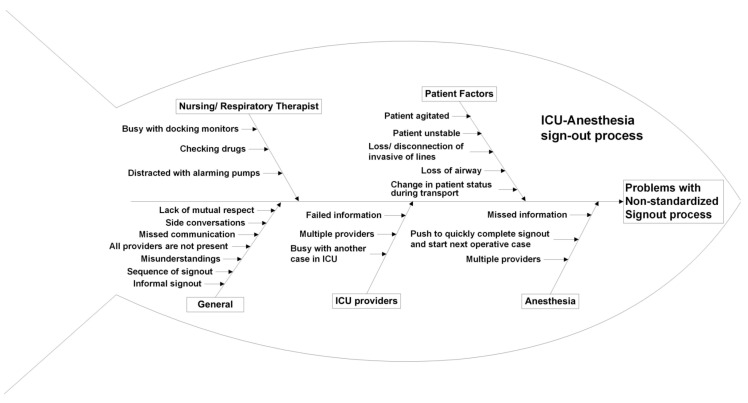
Fish bone diagram showing the causes of the problems contributing to an inefficient/ineffective hand-off process between the anesthesia and operating room teams.

A multidisciplinary team of experts met to discuss the existing hand-off process during the transition of care of children from the NICU and PICU. The team included stakeholders from anesthesiology, NICU, PICU, surgery, respiratory therapists and nursing staff. A baseline pre-implementation survey (Survey 1) was conducted in order to determine the provider perceptions of the current practice of hand-off and need for any improvements. It was identified that no standardized hand-off process or tool existed. Following multiple meetings and discussions, the team identified various problems and factors contributing to the variability in the hand-off process. Please refer to the fishbone diagram in Figure 1 for details. 

It was identified that a structured hand-off process was needed in order to improve communication amongst staff members and providers. This was designated as the TIME OUT FOR SIGN OUT (TOSO) process based on the Joint Commission “time-out model” as the universal protocol for the start of a procedure [1].

**Figure 2:**
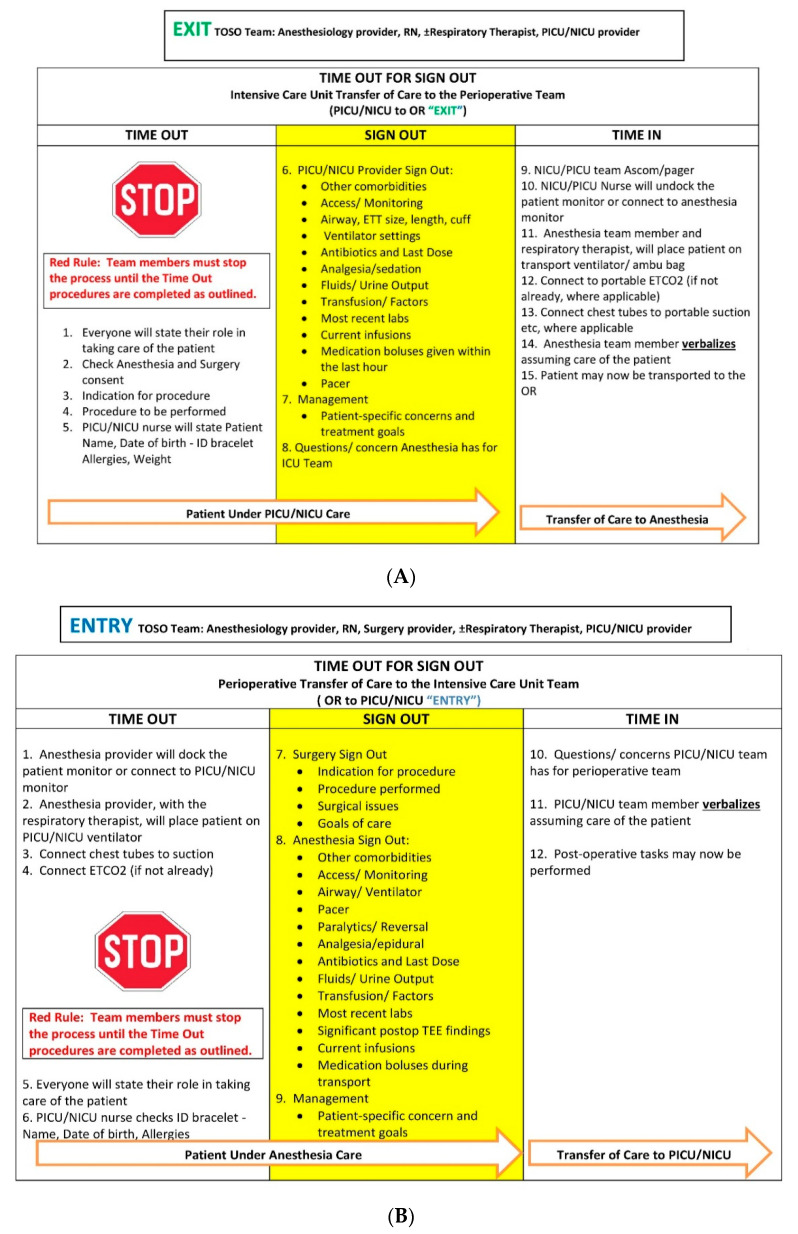
(**A**). Exit intensive care unit (ICU), i.e., hand-off from ICU teams to anesthesia team when transporting child to the OR. (**B**). Entry ICU, i.e., hand-off from anesthesia and surgery teams to ICU teams when transporting child from operating room (OR) to ICU.

This process entailed a formal interactive sign out between teams, in a standardized sequence, after ensuring that the patient was stable and all members of the multidisciplinary team were able to participate. TOSO was applied to both the entry into and exit from ICUs for all children transported by an anesthesia team. Posters were designed as a prompt to help facilitate the interactive processes (Figure 2A,B). Additionally, a write-on paper tool (prompt form) including important clinical details to be communicated (filled out by the provider initiating the hand-off) was designed to assist providers during the process [7]. This has been described elsewhere [7]. 

Please see the Appendix A for the prompt form used during the study. 

### 2.3. Assessing the Intervention

We performed staff satisfaction surveys and performance audits of our process in order to monitor and evaluate our intervention during the timeline from November 2015 to February 2019. Our QI project was geared towards the deficiencies and inconsistencies with the prior hand-off process between the ICUs and ORs. The pre-implementation survey was used to identify issues we needed to improve as well as provide a marker for satisfaction and improvement in our quality project. The post-implementation surveys identified whether providers felt the process had enhanced patient hand-offs. The performance audit was geared towards identifying the process of the hand-off and whether any critical elements were missed.

#### 2.3.1. Survey

In order to evaluate the impact of our intervention on staff satisfaction, we conducted an online survey, using SurveyMonkey (Copyright^©^ 1999–2019 SurveyMonkey), open to all providers (anesthesia, surgery, nursing, intensivist, and respiratory therapist teams) involved in the care of children during the hand-off process in both NICUs and PICUs. The survey consisted of eight questions that included general data including provider roles, workplace unit and years of experience, as well as pertinent questions about the process. For survey Questions 4, 5, 6 and 7, a qualitative scale of strongly agree, agree, neutral, disagree, strongly disagree was used. The survey was conducted in three phases: pre-implementation/Survey 1 (November–December 2015), six-month post-implementation/Survey 2 (July–August 2016) and >2 year follow-up post-implementation/Survey 3 (June–July 2018).

The survey questions are listed below:

(1) What Unit do you work in?
(2) What is your role?
(3) How many years have you worked in your current unit?
(4) The time out for sign out process provides the necessary information about the patient.
(5) The sign out system makes it comfortable for you to ask question to the OR/ICU team members.
(6) The sign out system is efficient and limits needless information about patient care.
(7) At the end of the time out for sign out, do you feel like the sign out was a necessary and important part of patient care?
(8) Do you feel the time out for sign out can be improved?

#### 2.3.2. Audit

In order to evaluate the TOSO performance, the clinical head nurse for the pediatric or neonatal ICU silently observed the interaction of the hand-off process during the pre-implementation and post implementation phases. The number of encounters audited was based on the availability of the clinical head nurse (random convenience sample). This was also conducted in three phases—pre-implementation/Audit 1 (November–December 2015), six-month post-implementation/Audit 2 (post-implementation January–June 2016) and >2 year follow-up post-implementation/Audit 3 (November 2018–February 2019). The data collected from the audit included: (1) whether a hand-off was completed, (2) critical items covered as part of the hand-off and (3) the hand-off time (time in minutes from the verbalization of initiation of hand-off to the verbalization of completion and dispersion of the team). Based on an expert consensus, the six items that were considered important for discussion at the hand-off included: airway/ventilation, intravenous access, medications administered, pertinent laboratory values, pertinent pre-operative (e.g., previous failed intubation, failed extubation) and pertinent intraoperative events (issues with ventilation intraoperatively, problems with intubation, need for a vasopressor, etc.).

### 2.4. Statistical Analysis

Sigma plot software (SigmaPlot^®^ 12.5 Systat Software Inc., San Jose, CA, USA) was used for analysis of statistical data. The general data were expressed as numbers and percentages. The Chi square test was used to test the significance of data on proportion. The data for items missed and number of providers at hand-off process were analyzed using the Kruskal–Wallis one-way analysis of variance on ranks. A *p* value < 0.05 was considered significant.

## 3. Results

### 3.1. Survey Results 

Overall, the numbers of responses received were as follows: pre-implementation phase, 113 participants; six-month post-implementation survey, 85 participants, and >2 year post implementation survey, 135 participants.

The distribution of the results for individual survey questions 1–3 are as shown in Table 1.

The results of survey questions 4–7 are as shown as supporting material in Supplementary Figures S1–S4, respectively. A higher number of anesthesia providers participated in Survey 3 compared to Survey 1. A smaller proportion of participants had more than four years of experience in Survey 3 (43.2%) when compared to Survey 1 (51.3%) or Survey 2 (50%); however, this did not achieve statistical significance. There was an increase in the proportion of participants who either strongly agreed or agreed that the process had improved in providing necessary information (Question 4), making it comfortable to ask questions (Question 5), enhancing efficiency (Question 6) and was an important and necessary part of the hand-off process (Question 7), when comparing Survey 1 to Survey 3 (Table 2). In Survey 3, 49.2% of participants felt that the hand-off process could be improved versus 78.4% in Survey 1 and 54.2% in Survey 2 (*p* < 0.001). 

**Figure 3:**
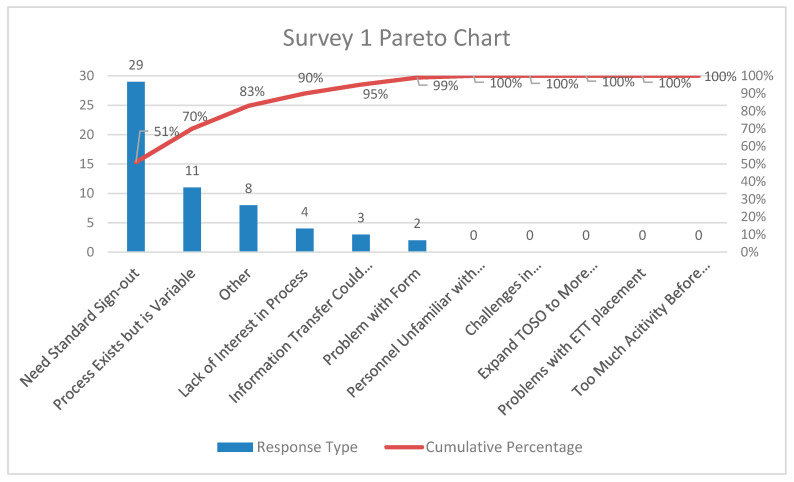
Pareto chart for comments from providers—Survey 1(pre-implementation survey).

**Figure 4:**
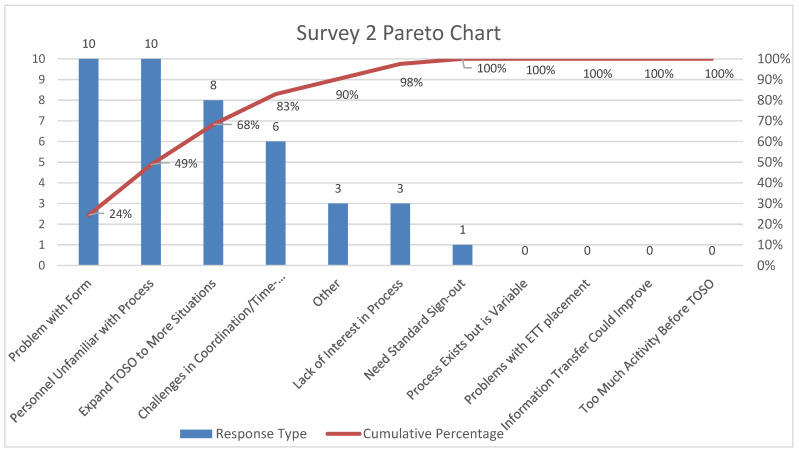
Pareto chart for comments from providers—Survey 2 (six-month post-implementation survey).

**Figure 5:**
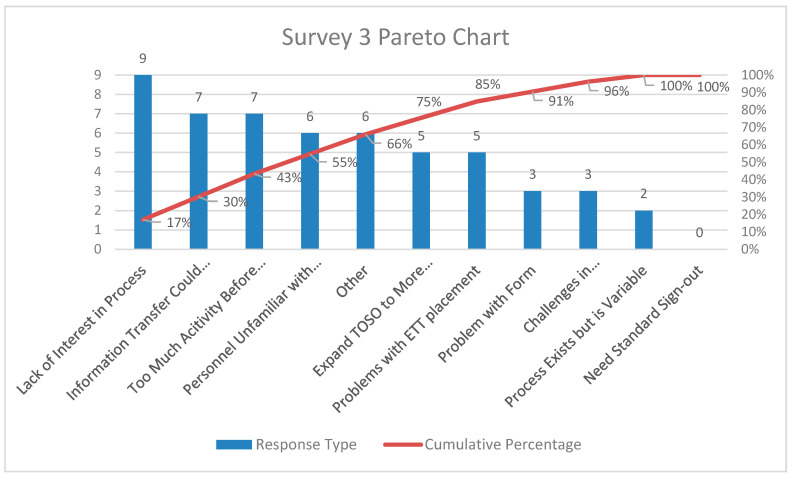
Pareto chart for comments from providers—Survey 3 (>2 years post-implementation survey).

Pareto charts of the comments from participants for the three surveys are shown in Figure 3, Figure 4 and Figure 5, respectively. There was a decrease in the proportion of provider comments with regards to the need for a formal hand-off process after implementation. There was an increase in comments related to expanding the project to other areas including the post-anesthesia care unit and the intermediate care unit following implementation. 

### 3.2. Audit Results

Overall, the number of hand-offs audited were 27 for Audit 1 (pre-implementation), 55 for Audit 2 (6 months post-implementation) and 38 for Audit 3 (>2 years post-implementation). The results of the three audits are as shown in Table 3.

The process timelines for the entire quality improvement project are shown in the supporting information additional figures—Supplementary Figure S5.

## 4. Discussion

Transfer of a patient between the OR and ICU entails two phases: a hand-off process and a physical transfer. The hand-off process is an important part of patient care because an inadequate hand-off can compromise patient safety. The Joint Commission recommends five components to be included in a hand-off. These are represented by the acronym “SHARE”: standardize, hardwired, ask questions, reinforce (quality), and educate [8]. The principles of an ideal hand-off process are: (1) a real-time face-to-face process, (2) patient-specific, (3) standardized, (4) structured, (5) interactive, (6) conducted with minimal interruption, (7) communicate patient updates, (8) opportunity to ask questions, (9) anticipated plan and (10) ability to easily contact the team signing out following dispersion after hand-off [9]. We have applied these principles by implementing an interactive hand-off process or time out for sign out (TOSO).

In the pre-implementation phase, we identified several problems including the lack of a standardized process, missed information, gaps in communication, as well as limitations with follow-up communication and information ownership. Following detailed meetings of stake holders, literature searches, and identifying the above problems, we standardized our hand-off process to address these concerns. A key component of our TOSO was the interaction between the teams to ensure a detailed hand-off and transfer of responsibility. Further, our interactive process ensured that (1) the hand-off process commenced only after ensuring the stability of the patient, (2) all participants were present during the process, and (3) there was clear verbalization of the transfer/acceptance of responsibility.

We followed our pre-implementation baseline survey with a post-implementation survey at 6 months after implementation, and another survey >2 years after implementation. Overall, staff felt there was an improvement in the transfer of information, comfort in asking questions, and efficiency and importance of the hand-off process following the institution of a formal process. Our surveys were useful in assessing our process change from the individual provider’s perspective. We followed up with an additional post-implementation survey to evaluate the continued success of our QI process. Across the three surveys, there was a decrease in the number of providers who felt that the process could be improved. Based on provider comments, identified areas for improvement included expanding the hand-off process to other areas such as the post-anesthesia care unit or intermediate care unit. 

Following the successful implementation of our quality project in children’s ICUs, our TOSO process for “Exit” and “Entry” ICU has been expanded to adult ICUs throughout the hospital and designated the “universal Time out for sign out process” [7]. Although information on the hand-off tool for each ICU may differ, we endeavored to have pertinent information on the hand-out paper tool, with a minimal risk of losing important information [7]. Our emphasis was on the “interactive” processes of communication rather than a paper tool which can be available for later referral by the ICU team. Although a universal TOSO for all ICUs, including adult ICUs, appears to be appropriate and useful, there is a risk of losing specific pediatric-related information. Perhaps a general adaptability of the paper tool may not be feasible, although the principles of TOSO are still applicable [7].

The model that we used for our standardized process is similar to that reported by other quality investigators in the ICU setting [3]. We involved stake holders from relevant disciplines who were responsible for disseminating the initiation, project progression, implementation, staff education and communication for their respective disciplines. In contrast to another study [3], we have demonstrated a significant improvement in each of the survey question items (4, 5 and 6; provides necessary information, comfortable to ask questions and efficient). Further, our survey results represent multidisciplinary providers and includes a comprehensive team of physicians (anesthesia, intensivist, and surgeon), nurses and respiratory therapists (not included in other studies). 

A previous study [10] utilized expertise from Formula 1 and aviation industries to improve patient handover in the operating room–ICU setting in a children’s hospital. The investigators noted that a great variability in the health care setup work force has potential for missed information, miscommunication, staff dissatisfaction and unfavorable outcomes. 

A structured handover process for pediatric cardiac surgery has been described, involving a pre-handover form filled out by an OR nurse and sent to the pediatric ICU via a pneumatic pod system, allowing staff to anticipate and be appropriately ready when they receive the patient [11]. The authors found an improvement in transferring the information of 19 essential points [11]. This finding is similar to our study where we observed a decrease in the missed critical items at the time of hand-off, following the implementation of the standardized process. We focused on the interactivity of our standardized hand-off process, reiterating the value of communication, leadership and teamwork, considered as nontechnical skills contributing significantly to human errors [12,13,14]. Our standardized process incorporates all these aspects of a hand-off that begins with the pre hand-off (nurse calls the ICU for a hand-off over phone informing the current status) followed by an interactive standardized hand-off when all parties are present, the patient is stable, monitors are docked and there are no distractions. Our longitudinal analysis demonstrates the continued staff satisfaction and improvement while identifying areas for further improvement. This sustainability is in keeping with another study that demonstrated the continued efficacy of a standardized handover in a pediatric cardiac ICU [15]. 

Our study reports an improved staff satisfaction in the post-implementation phase similar to a previous study [3] that reported an improved satisfaction of staff and clarity regarding the start and end of handover process [3]. The TOSO was at the cost of no additional time, yet was a succinct process.

### Study Limitations

The survey relied on self-reporting by the providers. There was observer bias with regard to conducted audits. The number of encounters audited were based on the availability of the auditor (clinical head nurse). The inter-rater reliability of the auditors was not tested. A standardized audit process could have reduced observer variability and bias. However, this needs more resources and warrants future studies in this direction. Further, we have not studied the interactions between providers’ professional and mutual respect at hand-off. This would warrant detailed research with standardized protocols and video clips to assess interactions. Ours was a QI project aimed at standardizing and improving our hand-off process. Improved patient outcomes, reductions in morbidity and decreases in missed information rates would be more suitable markers to judge the efficacy and success of the standardized interactive hand-off process. Although, improved outcomes have been reported in one study [16], these outcome measures have several confounding variables not taking into account patient comorbidities, observer bias and other associated causes.

## 5. Conclusions

The results of our QI project demonstrate an overall improved efficiency, satisfaction and sustainability of the standardized interactive hand-off process for the transfer of care between children’s ICUs and operating rooms. Perhaps it may serve as a model for other institutions.

## Figures and Tables

**Table 1:** Demographic data for provider responses to survey questions 1, 2 and 3, NICU = Neonatal Intensive Care Unit, PICU = Pediatric Intensive Care Unit, CRNA = Certified Registered Nurse Anesthetist, CRNP= Certified Registered Nurse Practitioner, PA = Physician Assistant. Results are expressed as percentages, values are rounded up.

Survey Questions	Answer Options	Survey 1 Response % *n* = 113	Survey 2 Response % *n* = 85	Survey 3 Response % *n* = 135	*p*-Value, Chi-Square Test
**Question 1, Unit of the Provider**	Anesthesia	27	29	40	*p* = 0.258
	NICU	38	38	28	
	Pediatric Surgery	3	1	4	
	PICU	32	32	28	
**Question 2, Provider role**	Nurse	40	53	23	*p* < 0.001
	CRNP/PA	6	7	8	
	Attending Physician	21	22	23	
	CRNA	4	1	11	
	Resident/Fellow	16	17	22	
	Respiratory Therapist	13	0	13	
**Question 3, Provider Experience**	<2 years	19	29	26	*p* = 0.43
	>2 years to <4 years	30	21	31	
	≥4 years	51	50	43	

**Table 2:** Percentage of participants who agreed or strongly agreed.

Survey Question	Survey 1 *n* = 113	Survey 2 *n* = 85	Survey 3 *n* = 135	*p*-Value, Chi Square Test
Question 4 (Provides Necessary Information), %	40.5	90.5	89.5	<0.001
Question 5 (Comfortable to Ask Questions), %	47.8	75.3	83.7	<0.001
Question 6 (Efficient and Limits Needless Information), %	30.4	64.7	70.6	<0.001
Question 7 (Necessary and Important), %	65.1	79.7	80.7	0.237

**Table 3:** Results of the audit regarding missed information at hand-off, IQR = Interquartile range.

	Audit 1 (Pre-Implementation) *n* = 27	Audit 2 (Six Months Post-Implementation) *n* = 55	Audit 3 (>2 Years Post-Implementation) *n* = 38	*p*-Value, Chi-Square Test
Formal Hand-off Process Occurred, %	88.8	91	94.7	0.08
Full Completion of Hand-off, %	44.4	87.2	81.5	<0.001
Items Missed during Hand-off, %	22	9	5	<0.001
Laboratory Values Information Missed, *n* (%)	10 (37)	7 (12.5)	6 (15.7)	0.032
Airway Ventilation Information Missed	5 (18.5)	5 (8.9)	1 (2.6)	0.09
Medication Information Missed	5 (18.5)	5 (8.9)	3 (7.8)	0.339
Time for Completion of Hand-off, Median, IQR; Minutes	5 (3, 8)	5 (3.75, 7)	6 (5, 9.75)	0.251

## Supplementary Materials

The following are available online at https://www.mdpi.com/2227-9067/7/9/123/s1, Figure S1: Distribution of responses for Survey Question 4—the time out for sign out process provides the necessary information about the patient. Figure S2: Distribution of responses for Survey Question 5—the sign out system makes it comfortable for you to ask question to the OR/ICU team members. Figure S3: Distribution of responses for Survey Question 6—the sign out system is efficient and limits needless information about patient care. Figure S4: Distribution of responses for Survey Question 7—at the end of the time out for sign out, do you feel like the sign out was a necessary and important part of patient care. Figure S5: Timelines for the Quality Improvement (QI).

## Appendix A

Exit and Entry ICU (Intensive Care Unit) prompt forms, NICU = Neonatal Intensive Care Unit, PICU = Pediatric Intensive Care Unit, ETT = Endotracheal tube IV = intravenous, FiO2 = Fractional inspired oxygen concentration, Paw = Peak airway pressure, TV = tidal volume, PS = pressure support, Hct = hematocrit.

**EXIT-ICU: NICU/PICU to Anesthesia Transfer of Care: Time out for Sign out Tool**

*(not a part of medical record)*

Name         DOB         MR #         Procedure

1. Comorbidities
2. Access—peripheral         central venous         arterial line
3. ETT/tracheostomy-size         length         cuffed/uncuffed
4. Ventilator settings
  FiO2     Mode     Paw     TV     PS     PEEP
5. IV infusions
6. Medications due for Operating room
7. Availability of blood/blood products
8. Last set of labs
  Hb/Hct                  Blood glucose
9. Post-operative plan for ventilation
10. Any issues/concerns
  ICU attending, contact #           ICU resident contact #

**ENTRY ICU: Anesthesia Team to NICU/PICU Transfer of Care: Time out for Sign out**

*(not a part of medical record)*

Name         DOB         MR #         Procedure

1. Airway ETT        size        type       length       issues
2. Recommended Ventilation settings, FiO2   Paw    TV   Ventilation mode   PEEP
3. New access established in OR
4. Anesthetic agents used
5. Last dose of narcotics, anesthetic agent, sedative or neuromuscular blocking agent
6. Analgesics in OR—acetaminophen, narcotics, regional block and infiltration of surgical wound
7. Reversal agents administered
8. If epidural—plan, drug, infusion rate
9. Antibiotics administered in OR, dose and time
10. IV infusions—inotropes, dosage rate
11. Total fluids administered
12. Blood or blood products                Estimated blood loss
13. Intraoperative labs                     blood glucose
14. CP bypass,                    times
15. Any specific issues and concerns
  Attending Anesthesiologist contact #       Anesthesia Resident contact #
