# Supplementary material for: A Model for a Standardized and Sustainable Pediatric Anesthesia-Intensive Care Unit Hand-Off Process
Source: Children (Basel). 2020 Sep 3;7(9):123. doi: 10.3390/children7090123 (PMC7552720; doi:10.3390/children7090123)
Supplement: Supplementary file 1 [file children-07-00123-s001.pdf]

Supplementary Materials: S1-S4, Figures for Survey Questions 4-7; S5, Time line chart for the Quality Project

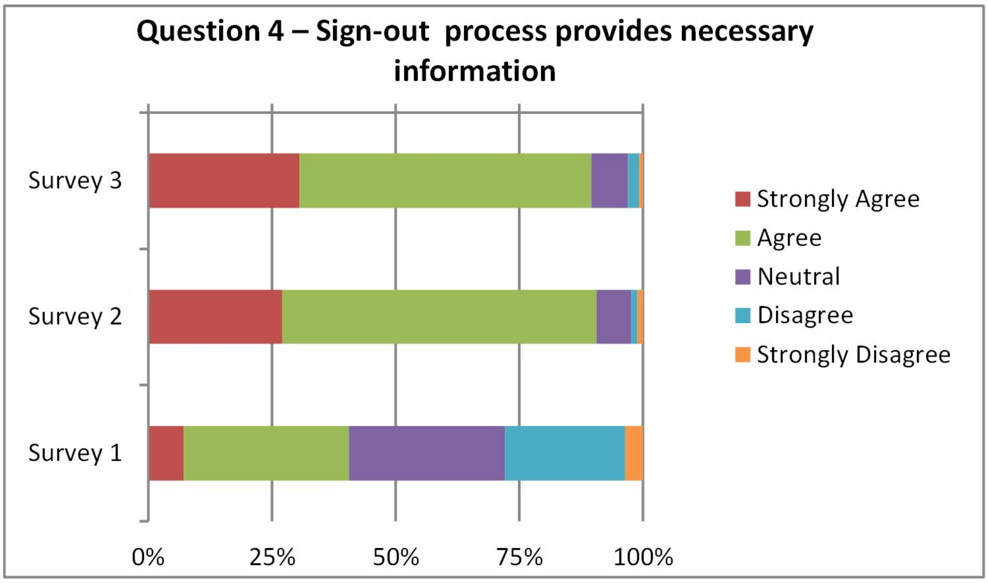

Figure S1: Distribution of responses for Survey Question 4—the time out for sign out process provides the necessary information about the patient.

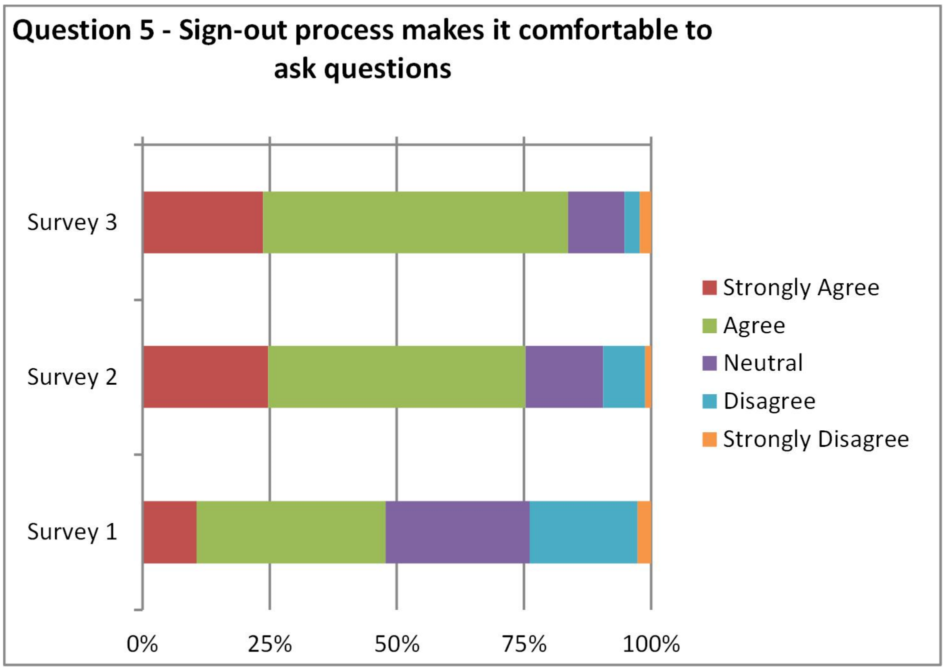

Figure S2. Distribution of responses for Survey Question 5—the sign out system makes it comfortable for you to ask question to the OR/ICU team members.

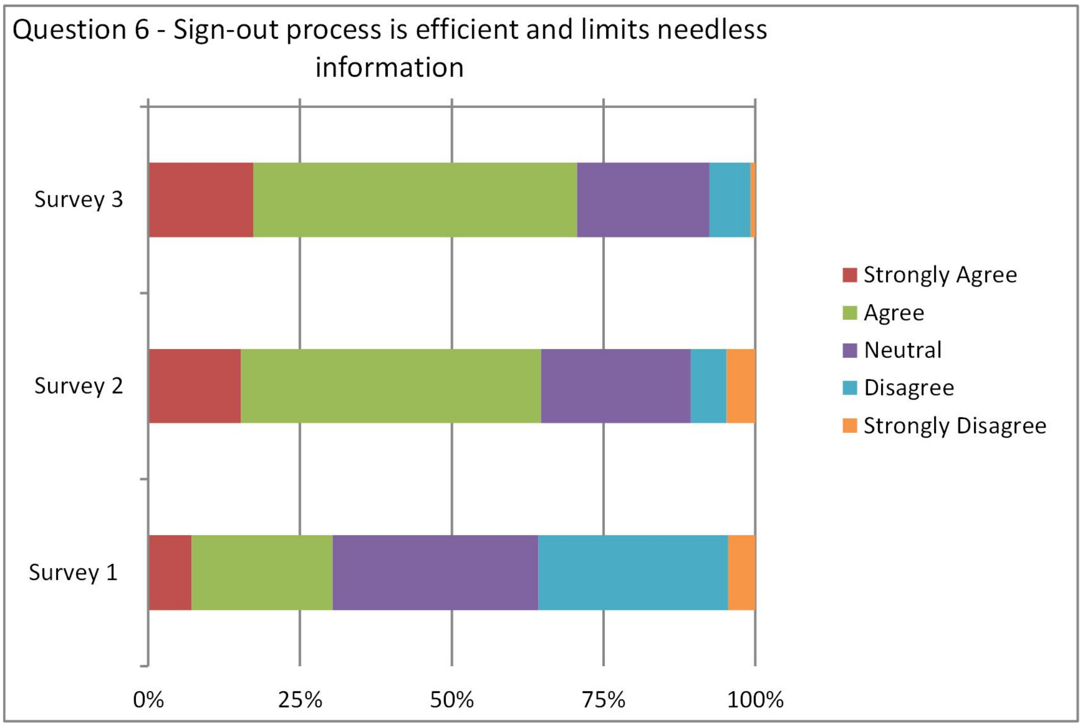

Figure S3. Distribution of responses for Survey Question 6—the sign out system is efficient and limits needless information about patient care.

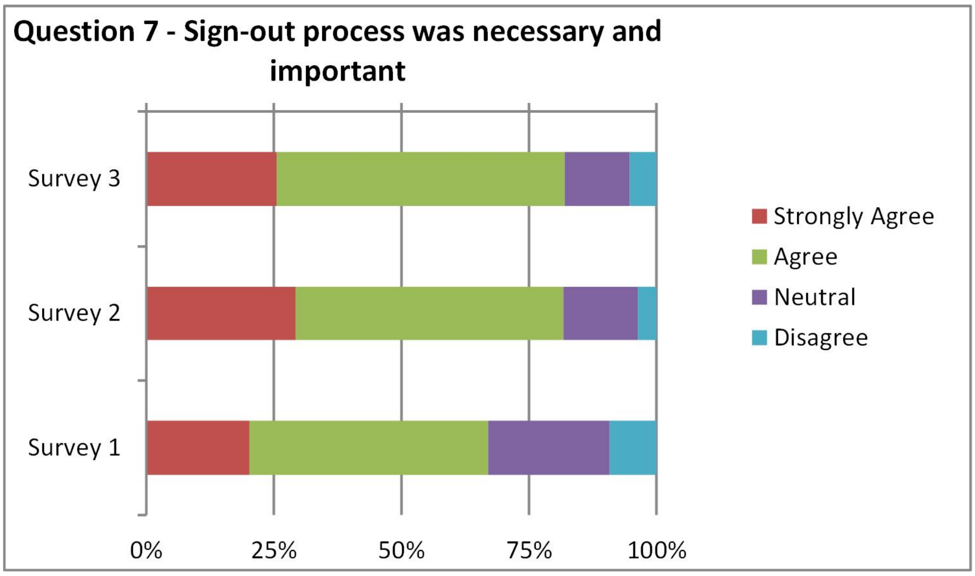

Figure S4. Distribution of responses for Survey Question 7—at the end of the time out for sign out, do you feel like the sign out was a necessary and important part of patient care.

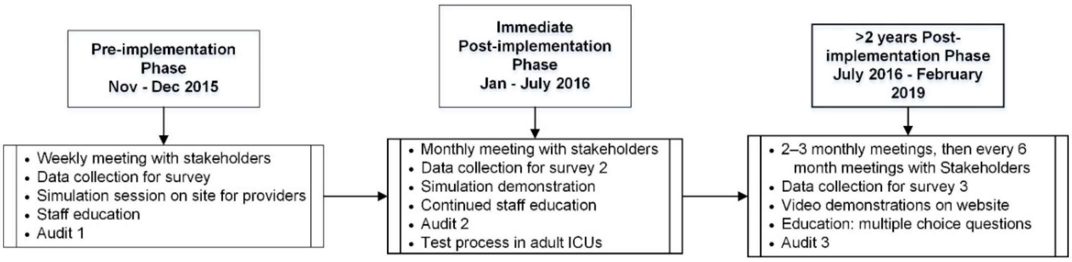

Figure S5. Timelines for the Quality Improvement (QI).
